# Patients with Gastrointestinal Bleeding and Atrial Fibrillation: Potential Ideal Target for Epicardial Appendage Occlusion

**DOI:** 10.3390/jcdd12050173

**Published:** 2025-05-01

**Authors:** Stefano Branzoli, Massimiliano Marini, Domenico Catanzariti, Cecilia Pravadelli, Luigi Pannone, Giovanni D’Onghia, Mauro Fantinel, Fabrizio Guarracini, Gaia Franceschini, Mirco Zadro, Giulia Baroni, Silvia Casagrande, Donatella Ottaviani, Renato Turco, Serena Nicolussi Paolaz, Luciano Annicchiarico, Francesco Corsini, Roberto Rordorf, Kausilia Krishnadath, Flavia Ravelli, Carlo de Asmundis, Mark La Meir

**Affiliations:** 1 Cardiac Surgery Unit, Santa Chiara Hospital, Largo Medaglie d’Oro, 38122 Trento, Italy; mark.lameir@uzbrussel.be; 2 Cardiac Surgery Department, Universitair Ziekenhuis, Av du Laerbeek 101, 1090 Brussel, Belgium; 3 Department of Cardiology, Santa Chiara Hospital, Largo Medaglie d’Oro, 38122 Trento, Italy; massimiliano.marini@apss.tn.it (M.M.);; 4 Heart Rhythm Management Center, Universitair Ziekenhuis, Av du Laerbeek 101, 1090 Brussel, Belgium; 5 Department of Cardiology, Santa Maria del Carmine Hospital, Corso Verona 4, 38068 Rovereto, Italy; domenico.catanzariti@apss.tn.it; 6 Department of Gastroenterology, Santa Chiara Hospital, Largo Medaglie d’Oro, 38122 Trento, Italy; 7 Department of Cardiology, Santa Maria Hospital, Via Bagnols sur Ceze, 32032 Feltre, Italy; 8 Department of Cardiology, Niguarda Hospital, Piazza Ospedale Maggiore, 20162 Milano, Italy; 9 Cardiology Department, San Bassiano Hospital, Via Lotti, 36061 Bassano, Italy; 10 Neurology Unit, Santa Maria del Carmine Hospital, Corso Verona, 38068 Rovereto, Italy; silvia.casagrande@apss.tn.it (S.C.);; 11 Geriatrics Department, Santa Chiara Hospital, Largo Medaglie d’Oro, 38122 Trento, Italy; 12 Geriatrics Department, Santa Maria del Carmine Hospital, Corso Verona, 38068 Rovereto, Italy; 13 Neurosurgery Unit, Santa Chiara Hospital, Largo Medaglie d’Oro, 38122 Trento, Italy; luciano.annicchiarico@apss.tn.it (L.A.); francesco.corsini@apss.tn.it (F.C.); 14 Department of Cardiology, IRCCS San Matteo, Via Golgi, 27100 Pavia, Italy; 15 Gastroneterology Department, Universitair Ziekenhuis Antwerpen, 2650 Antwerp, Belgium; 16 Laboratory of Biophysic and Translation Cardiology, Department of Cellular Computational and Integrative Biology (CIBIO), Centre for Medical Sciences (CISMed), University of Trento, 38123 Trento, Italy

**Keywords:** atrial fibrillation, appendage occlusion, thoracoscopy, gastrointestinal bleeding, anticoagulants, antithrombotic therapy

## Abstract

Background: Gastrointestinal bleeding in patients with atrial fibrillation is an indication for left appendage occlusion. All endovascular devices mandate antithrombotic therapies: rebleeding risk remains an issue. To date, there are no reports on gastrointestinal rebleeding and stroke prevention by left appendage occlusion without any antithrombotic therapy in this category of patients. Methods: A total of 129 patients (male 85, mean age 76.6 ± 7.1, CHA_2_DS_2_Vasc 3.8 ± 1.5, HASBLED 3.3 ± 1.0; upper GI bleeding 10%, lower GI bleeding 86%, obscure occult 4.6%, on NOACS full dose 77.5%, NOACs reduced dose 13.1%, on anti-vitamin K 9.3%) with atrial fibrillation and history of repetitive gastrointestinal bleeding from ten centers underwent standalone thoracoscopic epicardial appendage closure without antithrombotic therapy for the entire follow up. Results: The observed bleeding rate was 0.91 events per year, equivalent to a relative risk of RR = 0.17 (*p* = 0.02) and a relative risk reduction (RRR) of 83%. The observed relative risk of stroke was 0.91 events per year, with a relative risk of RR = 0.19 (*p* = 0.03) and a relative risk reduction (RRR) of 81%. Conclusion: Standalone epicardial appendage occlusion without antithrombotic therapy in patients with repetitive gastrointestinal bleeding is safe and promising when rebleeding and stroke risk reduction need to be optimized.

## 1. Introduction

Atrial fibrillation (AF) is the most common arrhythmia and has significant clinical and social burden with a concomitant economic impact [1,2]. Despite the net benefit of NOACS over anti vit K anticoagulants, no better outcomes in terms of gastrointestinal bleeding (GIB) have been reported, even with reduced doses, and low adherence to NOACs has been found to be associated with a higher risk of thromboembolic events compared to high and low adherence to anti vit K [3,4,5]. A lifelong oral anticoagulation effect may be difficult to manage in an aging population with increased frailty [5,6]. From the paradigm of the guidelines to everyday practice, left atrial appendage occlusion (LAAO) has become an accepted therapeutic option that is frequently offered to patients [1,2]. Along with cerebral hemorrhage, a history of GIB is a common indication for standalone LAAO performed either transcatheter endocardially or thoracoscopically via epicardial access (TT-LAAO) [1,2]. To date, there are only two reports on the safety and efficacy in terms of stroke and hemorrhage prevention of LAAO in patients with recurrent GIB: both used endovascular devices [7,8]. The mandatory early, post-procedural more aggressive and subsequent lifelong less aggressive antiplatelet therapy required by all endovascular devices is still subject of active debate; this due to the presence of a foreign body in a low-flow cardiac chamber [9,10]. Bleeding occurrence in the short, middle and long terms remains a clinical issue with any antithrombotic regime, and even more so in patients referred for LAAO due to chronic GIB not amenable to definitive treatment [1,11,12,13,14]. Data on the clinical efficacy and safety of LAAO without any antithrombotic therapy in this category of patient is missing. LAAO with an epicardial device “per se” not requiring any antithrombotic therapy intra/post procedurally potentially represents a valid therapeutic option but has never been investigated. Here, we present our results on the largest series of patients eligible for LAAO due to recurrent GIB who underwent totally thoracoscopic epicardial appendage occlusion without intra- and post-procedural antithrombotic therapy for the entire follow-up.

## 2. Patients and Methods

### 2.1. Patient Selection

The study was conducted in accordance with the Declaration of Helsinki, was approved by the Hospital Ethics Committees and the data were collected accordingly for privacy data protection (A379 approved on 2 May 2017). Written informed consent was obtained from all patients.

As a multicenter collaboration, patients were enrolled in the departments/units of ten Hospitals.

Inclusion criteria: AF and history of recurrent GIB confirmed at endoscopic, videocapsule, angiography, angio-CT.

Exclusion criteria: previous left lung surgery, life expectancy < 1 year.

After multidisciplinary evaluation and discussion of all available alternative options with the patient, 129 patients were referred to TT-LAAO in the period 2017–2023. Two patients denied consent for self-perceived frailty.

Patient characteristics are presented in Table 1 (male 64.8%, mean age 76.6 ± 7.2, mean CHA_2_DS_2_Vasc 3.8 ± 1.5, mean HASBLED 3.3 ± 1.1, causes of GI bleeding (upper 10%, lower 86%, 4.6% unknown site), number of patients with previous transfusion 82.1%, mean number of admission for blood transfusion/pt 2.1 ± 1.5, four patients with i.v. iron supplement due to units of blood refusal, mean number of invasive diagnostic tests/patient was 2.5 ± 1.9).

### 2.2. Etiology and Severity of Bleeding

**Figure 1:**
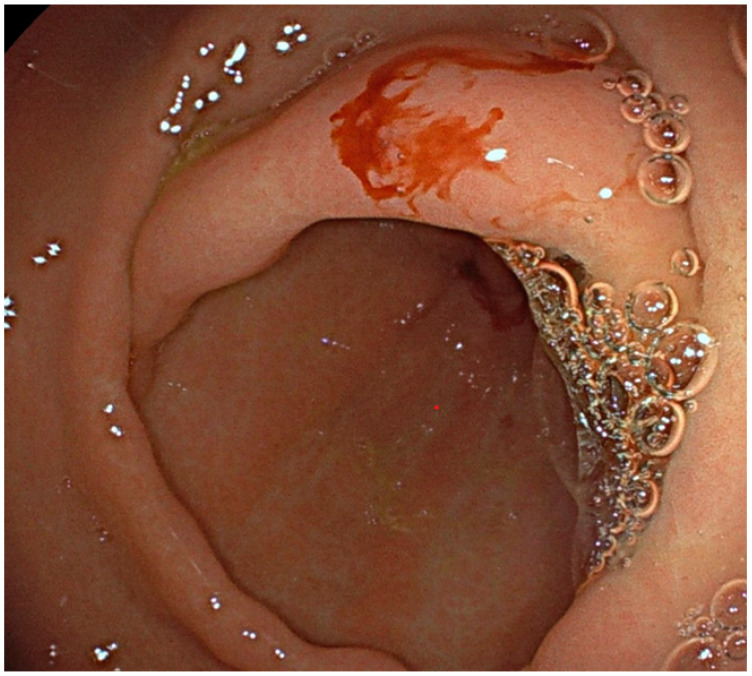
Bleeding Angiodysplasia.

Significant GIB was defined as drop of >2 g/dL of hemoglobin for occult bleeding or by documentation of melena, hematochezia, hematemesis, rectal bleeding, bleeding stigmata confirmed by a physician and by complete endoscopic/radiologic evaluation excluding any treatable source of bleeding (Figure 1).

Bleeding was classified as upper or lower if proximal or distal to the ligament of Treitz, respectively, and unknown if no apparent source was found (all other causes of non-GI bleeding were excluded, i.e., obscure occult bleeding) after full endoscopic evaluation or when the patient refused to undergo a further diagnostic procedure. Bleeding was classified in overt, obscure and occult in accordance with European Endoscopy Society [13,14]. Bleeding Academic Research Consortium (BARC) criteria were used for bleeding type. BARC bleeding was classified into type 0, no evidence of bleeding; type 1, bleeding that does not cause the patient to seek clinical care; type 2, bleeding that requires diagnostic studies, hospitalization or treatment; type 3, overt bleeding requiring a blood transfusion or with hemoglobin drop > 2.0 g/L; type 4, coronary artery bypass graft-related bleeding; and type 5, fatal bleeding [15]. Only patients in BARC 2–5 were included and only after all correctable/modifiable factors were assessed and optimized before proceeding to LAAO. A 7 g/dL threshold for transfusion was set but not consistently respected among centers as the final decision, which was left to the referring physicians and based upon individual patient frailty and comorbidities.

### 2.3. Study Outcomes

The widely accepted definition of technical success as a residual stump < 1 cm at TOE/CT scan was used [16]. The primary outcomes considered were all bleeding, re-GI bleeding requiring transfusion or Hgb drop > 2 g/dL, stroke/TIA, embolic events and procedure/device related death. The secondary outcomes were hospital readmission that was procedure/device related, infection, pneumothorax, pleural effusion, dehiscence, pericarditis and periprocedural bleeding.

### 2.4. Statistical Analysis

All variables were tested for normality using the Shapiro–Wilk test. Normally distributed variables were described as mean ± standard deviation and the groups were compared through ANOVA or paired or unpaired *t*-test as appropriate, while the non-normally distributed variables were described as median (Inter Quartile Range) and compared via Mann–Whitney or Wilcoxon signed-rank tests as appropriate. The categorical variables were described as frequencies (percentages) and compared using the Chi-square test or Fisher’s exact test as appropriate. Relative risk (RR), relative risk reduction (RRR), number needed to treat (NNT) and relative confidence intervals were calculated.

Kaplan–Meier survival analysis was performed to analyze the cumulative event rates.

Cox’s proportional hazard model was not performed because of the low event rate.

A *p*-value less than 0.05 was considered statistically significant.

The analysis was performed using R software version 3.6.2 (R Foundation for Statistical Computing, Vienna, Austria) and SPSS Statistics 23.0 (IBM Corp, Armonk, NY, USA).

### 2.5. Follow-Up

All patients underwent full clinical examinations at 1, 2, 6 and 12 months and yearly thereafter, including the filling in of the Questionnaire for verifying stroke-free status (QVSFS) as a screening tool validated by the Neurology Society [17] and a full blood test. An adjunctive full blood test was prescribed in case of a clinical suspicion of bleeding.

### 2.6. Preoperative Workup

The preoperative workup included chest X-ray, full blood exams, TOE for presence of thrombus in the LAA and CT scans in cases where there was a past medical history suggestive of an anatomical condition potentially interfering with left thoracoscopy or contraindication to TOE.

### 2.7. Procedure

**Figure 2:**
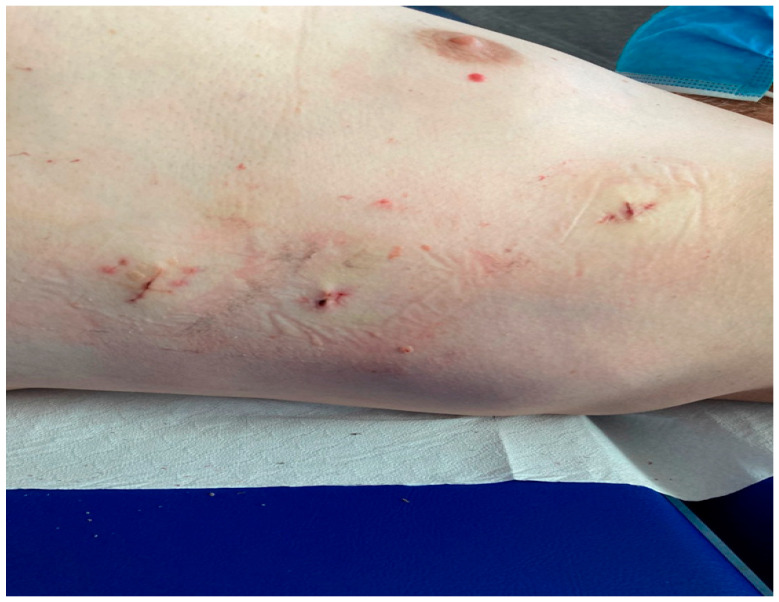
Thoracoscopic accesses “hockey stick”.

**Figure 3:**
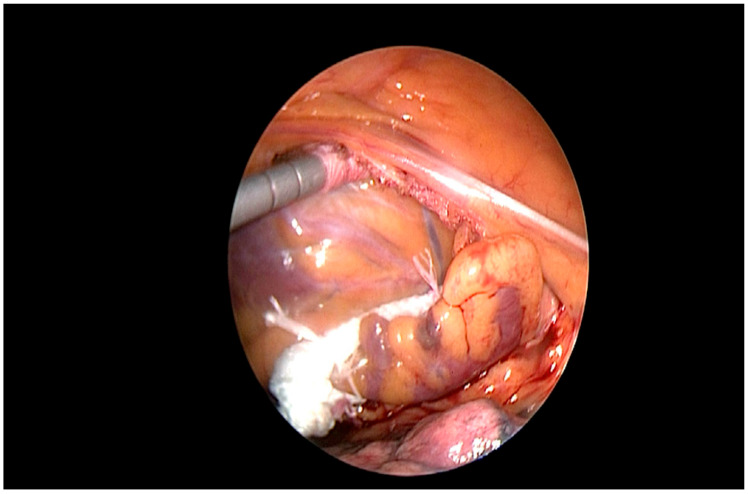
Epicardial closure.

The procedure was performed without heparin, as previously published by our group [18]: under general anesthesia, selective right lung ventilation 3 ports in III, V, and VII intercostal space between anterior and posterior axillary line in a “hockey stick” figure were placed (Figure 2), after CO_2_ insufflation (8 L/min, 8 mmHg pressure) and opening the pericardium the appendage was visualized, measured and occluded with the Atriclip Pro 2 or Pro V (ATRICURE, Mansion, OH, USA) under direct view and TOE monitoring in the absence of esophageal varices (Figure 3).

### 2.8. Post-Procedure Antithrombotic Therapy

No NOACs/anti Vit K/LMWH/DAPT/SAPT was prescribed from the day of surgery; two patients were prescribed DAPT during late follow-up after peripheral revascularization but the antithrombotic regime was interrupted when evidence of re-GI bleeding with Hb drop > 2 g/dL was documented. All patients were discharged on low-dose diuretics for some 20 days. Prophylactic colchicines/indometacine to prevent pericarditis was prescribed in 87% of patients.

## 3. Results

### 3.1. Procedural Success

Effective and successful LAA closure was documented in all patients. One patient with thrombus in the LAA received the PRO-V. At CT scan or TEE follow-up (37.9% and 62.1%, respectively), no residual LAA stump was reported in 92.2% of patients; the remaining patients had a stump of <1 cm (mean 3 ± 2 mm). The mean procedure duration and hospital stay were, respectively, 31.9 ± 13.6 min and 3.2 ± 0.7 days. All patients were discharged home and completed follow-up (mean 26.5 ± 19.5 months). The only complication reported was pericarditis in three patients, which was successfully treated at home pharmacologically. There were four deaths at 21, 22, 58 and 62 months that were not procedure/device related.

### 3.2. Clinical Success at Follow-Up

At a mean follow-up of 26.5 ± 19.5 months (median 24.2 months) (range 6–98 months), one TIA and one minor stroke were reported at 22 and 28 months in patients at higher risk of stroke and CHA_2_DS_2_Vasc compared with event-free patients.

Two patients required units of blood at 16 and 28 months despite no antiplatelet therapy, and two patients exhibited an Hb drop requiring transfusion while on dual antiplatelet prescribed following surgery for peripheral revascularization and experienced no further bleeding episodes after DAPT discontinuation. No statistically significant HASBLED score between patients with and without adverse bleeding events was found (Table 2).

**Figure 4:**
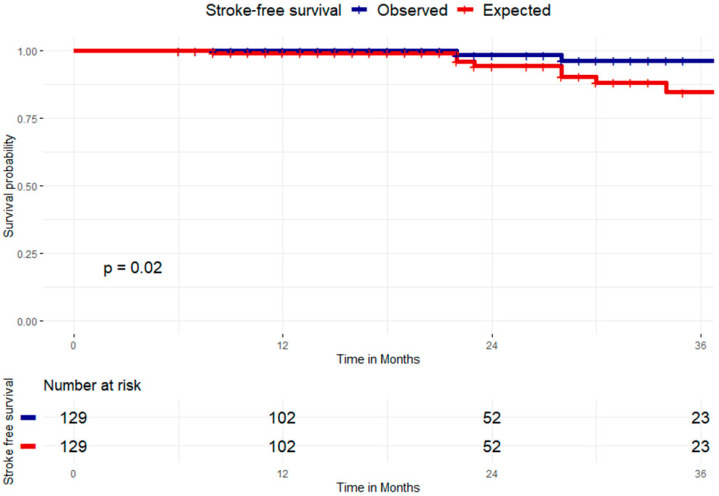
The observed relative risk of stroke was 0.91 events per year, corresponding to a relative risk reduction of RR = 0.19 (0.04–0.8), *p* = 0.03 and a relative risk reduction (RRR) of 81%.

The mean stroke risk per year (according to CHA_2_DS_2_VASC) was 4.5 events per year. The expected stroke events in the follow-up (2.2 years) was 9.9 events. The observed stroke rate was 0.91 events per year. This corresponds to a relative risk reduction of RR = 0.19 (0.04–0.8), *p* = 0.03 and a relative risk reduction (RRR) of 0.81. The number needed to treat was NNT = 15.8 (8.8–83.1) (Figure 4).

**Figure 5:**
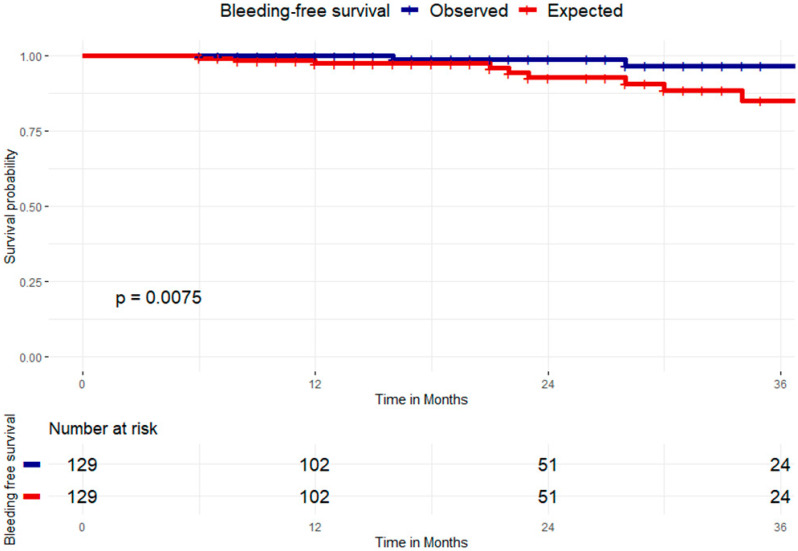
The observed bleeding rate was 0.91 events per year, equivalent to a relative risk of RR = 0.17 (0.03–0.73), *p* = 0.02 and a relative risk reduction (RRR) of 83%.

The mean bleeding risk was 5.5 per 100 patients per year (according to HASBLED score). The expected bleeding events in the follow up (2.2 years) was 12.1 events. The observed bleeding rate was 0.91 events per year. This corresponds to a relative risk of RR = 0.17 (0.03–0.73), *p* = 0.02 and a relative risk reduction (RRR) of 0.83. The number needed to treat was NNT = 12.9 (7.6–43.4), Figure 5.

## 4. Discussion

Managing patients with AF and recurrent GIB on anticoagulants is a clinical challenge. Endovascular LAAO with the mandatory post-procedure antiplatelet therapy has been investigated as a therapeutic option [7,8], but recurrent GIB remains an issue with any antiplatelet therapy [1,2,6,7,8,9,10,11,12,13,14,15]. To the best of our knowledge, this is the first study to investigate the potential benefit of epicardial appendage occlusion without any antithrombotic therapy in patients with AF and a history of repetitive GIB: a relative risk reduction of 83% and 81% for rebleeding and stroke, respectively, were found, with a concomitant high safety profile.

Atrial fibrillation requires oral anticoagulation, with NOACs used as first-line therapy over anti vit K [1,2]. Despite the several advantages of these molecules, no better outcomes in terms of GIB have been reported, even with reduced doses, and issues regarding low adherence remain [3,4,5]. According to guidelines, appendage occlusion is a treatment option for patients with contraindication/poor tolerance to long-term oral anticoagulation: repetitive GIB has therefore become an indication [1,2,5,7,8,16]. The prevalence of GIB in patients with AF has been estimated to be around 5.4% [19,20].

According to the TREAT AF and ORBIT AF studies, bleeding is the cause of anticoagulant discontinuation in up to 30% of cases, and GIB accounts for 8% of these [6]. Managing acute GI bleeding in patients on antithrombotic therapy is challenging, as is establishing the appropriate time for drug reintroduction [1,12,13]. Early resumption of anticoagulants/antiplatelet therapy is clinical practice, but this is based on studies with short-term outcomes that mainly show benefits for embolic prevention [1,12,13,19,20,21,22,23,24]; however, the outcomes for rebleeding and death are heterogeneous [19,20,21,22,23,24,25,26,27,28,29]. Specific guidelines do not provide unequivocal recommendations, and variable levels of evidence suggest a multidisciplinary consultation to guide the appropriate management of high-bleeding-risk patients [1,13,14,23,24,25,26,27,28,29].

The role of LAAO is as follows. Endovascular devices mandate lifelong antithrombotic therapy but, across trials and studies, there is no uniformity on prescription and time of progressive reduction with a wide range of different regimes [7,8,9,10] Notably, GIB pre-procedure has been shown to be an independent predictor for bleeding recurrence after endovascular device implantation and is also associated with an increased risk of death, supporting the hypothesis that patients experiencing GIB might benefit from the immediate suspension of antiplatelet therapy [7,8,11]. Minimum oral anticoagulation and dual antiplatelet therapy followed by long-term single antiplatelet therapy is usually recommended [1]. In a report by Han et al. aiming to evaluate GIB in patients at low risk of GIB on DAPT followed by SAPT, this regime was associated with a higher risk of gastrointestinal mucosal injury, whereas a simple SAPT regime was associated with a lower rate of gastrointestinal injury compared to DAPT [30,31]. GIB after transcatheter interventional cardiology is a common cause of non-access-site bleeding and has been associated with increased patient morbidity and mortality [26,30,32]. An extravascular device does not mandate antiplatelet therapy. Based upon this evidence we aimed to investigate the potential benefit of standalone thoracoscopic epicardial appendage occlusion in patients with AF and recurrent GIB on anticoagulants. In term of technical success, our findings are in line with published studies reporting success rates of 97.3–99.5% [7,8,33,34,35]. The incidence of pericarditis, the only complication documented, was in line with the literature and could be treated pharmacologically in all cases to complete resolution [33,34].

In terms of clinical success, the thoracoscopic approach with an RRR of 83% for bleeding compares favorably with Watchman (RRR 3% with 8.7 predicted vs. 8.4 observed considering the 4.3 in BARC 3–5 and 4.1 in BARC 1–2) and Amulet (RRR 64.8% excluding periprocedural bleeding and RRR 20.3% periprocedural bleeding included), having a comparable follow-up. In the Amplatzer Amulet Full Result Study, 6% of all patients had GIB (77.6% of all major bleedings) while on ANTI VIT K 10.5%, DAPT 41.8%, SAPT 34.3% or no therapy 13.4%. A report by Tarantini et al. on LAAO in patients with and without previous history of GIB also confirmed an increased rate of major bleeding for those patients with a positive history of GIB (13.2% vs. 4.7% *p* < 0.001). This led to a net benefit of the overall annual risk of major bleeding reducing by 39–44% compared to the predicted rate among subjects without history of GIB and increases of 53% and 67% in the major bleeding rate compared to those predicted in patients with previous GIB according to HAS BLED> and <3 [36,37].

In our study, only two cases of re-GIB were observed, both patients that had diffuse angiodysplasia; one underwent argon plasma coagulation and the other received close clinical monitoring and i.v. iron supplement. The two other cases of GIB were due to temporary DAPT reintroduction. The absence of any antithrombotic therapy in our study is in line with the seminal paper by Ohtsuka et al. [35] and differs from the papers on Watchman and Amulet, where at late follow-up the pharmacological regime was: none 18.4%, SAPT 62.5%, DAPT 11.4%, anti-vit K 1.3%, NOAC 4.8%, OAC + SAPT 1% and LMWH 0.6% for Watchman and 82.5% on aspirin, 22.4% clopidogrel, 1.4% warfarin and 0.7% NOAC for Amulet [7,8].

For cardioembolic events prevention, with an 81% RRR, our study further supports the efficacy of LAAO as a valid therapeutic option, which is in line with pertinent and relevant studies showing a 67–83% RRR [35,38], and confirms the safety of using no antithrombotic therapy post-procedure [33,35].

For endovascular devices, further investigation of potential device-related issues like PDL, DRT and appropriate post-procedure downgrading of antithrombotic therapy are needed [39]. To improve bleeding outcomes, there is a trend towards less aggressive prescription of antithrombotic therapy. For epicardial closure devices, there are now more data available to prove safety and efficacy and to show that post-procedure antithrombotic therapy is potentially not required [33,34,35,40]. Although randomized trials with long follow-up periods are missing, epicardial LAAO might be a reasonable option in specific categories of patients, such as those with a history of GIB that are unequivocally associated with rebleeding while on an antiplatelet regime [33,35,41]. The well-designed ASPREE trial, which had as an endpoint GI bleeding resulting in transfusion, hospitalization, surgery or death, showed an increased overall GI bleeding risk of 60% in healthy elderly patients on aspirin. The authors clearly demonstrated the implications of lifelong SAPT in frail patients eligible for endovascular LAAO [11].

### Limitations

The relatively small number of patients and absence of a control group are major limitations of the current study. The former is due to the limited diffusion of thoracoscopic cardiac surgery programs and lack of a strategic multidisciplinary approach to complex patients with atrial fibrillation and poor tolerance to anticoagulants. The second is due to the peculiar pathophysiology of repetitive GIB on antiplatelet therapy compared to other indications to LAAO and to the small number of patients in class BARC 1–2 enrolled to select a control group for meaningful conclusions. In addition, the reintroduction of an antiplatelet regime for comparisons in patients with documented repetitive GIB was judged not reasonable by the multidisciplinary team based upon the large amount of research available on this topic. The HAS BLED is a valid tool but it is imperfect, especially in cases of chronic recurrent bleeding. The relatively short follow-up, although comparable to other reports on this topic, may be another limitation, but the follow-up is ongoing.

## 5. Conclusions

Managing GIB in patients requiring antithrombotic therapy due to atrial fibrillation can be a major clinical challenge. Appendage occlusion is a therapeutic option and endovascular devices currently mandate at least lifelong single antiplatelet therapy with the risk of rebleeding, device-related thrombosis, and peridevice leak requiring introduction of more aggressive antithrombotic therapy. Epicardial closure might overcome these drawbacks of endovascular closure, but trials are required to improve our decision-making process and possibly the clinical outcomes. While waiting for the endovascular device with lowest prothrombotic profile, the optimal post-procedural antithrombotic therapy regime, proper DRT and PDL prevention, thoracoscopic LAAO appears a valid treatment option.

## Figures and Tables

**Table 1:** Patient demographics.

Variable	Value
Gender (M) (%)	85
Age (mean ± SD)	76.6 ± 7.1
CHA_2_DS_2_Vasc (mean ± SD)	3.8 ± 1.5
HAS BLED (mean ± SD)	3.2 ± 1
HASBLED ≥ 3 (%)	78.3
HASBLED < 3 (%)	21.7
EF (mean ± SD) (%)	53.5 ± 7.5
Previous stroke (%)	21.7
Previous cerebral Hemorrhage	5.4
Type of AF (%)	
Paroxismal	13.1
Persistent	5.4
Longstanding Pers	5.4
Permanent	75.9
BARC criteria (%)	
I	0
II	18.5
III	71.2
IV	0
V	10.1
Lower GI Bleeding (%)	86
Angiodysplasia (%)	70.4
Diverticular disease (%)	7.7
Inflammatory disease (%)	5.4
Hemorrhoids (%)	3.1
Upper GI Bleeding (%)	9.4
Liver failure, portal hypertension, esophageal varices	6.2
Gastroduedenal erosion	3.2
Obscure occult (%)	4.6
Anticoagulant regime before procedure	
Anti Vit K (%)	9.3
DOACs full dose (%)	77.5
DOACs reduced dose (%)	13.1
Number of transfusions pre-LAAO/Patient (mean ± SD)	2.1 ± 1.5
Number of invasive diagnostic procedures/patient (mean ± SD)	2.5 ± 1.9
**TOTAL NUMBER OF PATIENTS**	129

**Table 2:** Demographics of 125 event-free patients and 4 patients who experienced adverse events.

	No Stroke or Bleeding During Follow-Up (N = 125)	Stroke or Bleeding During Follow-Up (N = 4)	Total (N = 129)	*p* Value
Age	76.6 (7.2)	79.2 (3.9)	76.7 (7.2)	0.464
Gender (M)	83 (66.4%)	2 (50.0%)	85 (65.9%)	0.605
FE	53.6 (7.6)	51.2 (2.5)	53.5 (7.5)	0.538
CHADVASC	3.8 (1.5)	5.8 (2.1)	3.9 (1.5)	0.013
risk_stroke_year	4.4 (2.3)	7.4 (3.1)	4.5 (2.4)	0.012
HASBLED	3.3 (1.1)	4.0 (0.8)	3.3 (1.1)	0.185
bleeding_risk_year	5.4 (3.4)	8.4 (3.6)	5.5 (3.5)	0.084
transfusion_N	2.0 (1.5)	1.3 (0.9)	2.0 (1.5)	0.351
new_GRC	0.1 (0.4)	0.0 (0.0)	0.1 (0.3)	0.719
FUP	26.1 (19.1)	38.5 (22.8)	26.5 (19.2)	0.206
prevEC	7 (5.6%)	0 (0.0%)	7 (5.4%)	1.000
prev_stroke	25 (20.0%)	3 (75.0%)	28 (21.7%)	0.032
Diabetes	27 (21.8%)	1 (25.0%)	28 (21.9%)	1.000
Hypertension	69 (55.2%)	3 (75.0%)	72 (55.8%)	0.629
Death	4 (3.2%)	0 (0.0%)	4 (3.1%)	1.000

## Data Availability

The original contributions presented in this study are included in the article. Further inquiries can be directed to the corresponding author.

## Abbreviations

The following abbreviations are used in this manuscript:

AF	Atrial fibrillation
GIB	Gastrointestinal bleeding
LAA	Left atrial appendage
SAPT	Single antiplatelet therapy
NOACS	New oral anticoagulants
DAPT	Dual antiplatelet therapy
TOE	Transoesophageal echo
TTLAAO	Totally thoracoscopic appendage occlusion
